# Targeting Tregs in Malignant Brain Cancer: Overcoming IDO

**DOI:** 10.3389/fimmu.2013.00116

**Published:** 2013-05-15

**Authors:** Derek A. Wainwright, Mahua Dey, Alan Chang, Maciej S. Lesniak

**Affiliations:** ^1^The Brain Tumor Center, The University of ChicagoChicago, IL, USA

**Keywords:** malignant glioma, glioblastoma multiforme, regulatory T cells, Tregs, natural Tregs, tumor-induced Tregs, IDO (indoleamine 2,3-dioxygenase)

## Abstract

One of the hallmark features of glioblastoma multiforme (GBM), the most common adult primary brain tumor with a very dismal prognosis, is the accumulation of CD4^+^CD25^+^Foxp3^+^ regulatory T cells (Tregs). Regulatory T cells (Tregs) segregate into two primary categories: thymus-derived natural Tregs (nTregs) that develop from the interaction between immature T cells and thymic epithelial stromal cells, and inducible Tregs (iTregs) that arise from the conversion of CD4^+^FoxP3^−^ T cells into FoxP3 expressing cells. Normally, these Treg subsets complement one another’s actions by maintaining tolerance of self-antigens, thereby suppressing autoimmunity, while also enabling effective immune responses toward non-self-antigens, thus promoting infectious protection. However, Tregs have also been shown to be associated with the promotion of pathological outcomes, including cancer. In the setting of GBM, nTregs appear to be primary players that contribute to immunotherapeutic failure, ultimately leading to tumor progression. Several attempts have been made to therapeutically target these cells with variable levels of success. The blood brain barrier-crossing chemotherapeutics, temozolomide, and cyclophosphamide (CTX), vaccination against the Treg transcriptional regulator, FoxP3, as well as mAbs against Treg-associated cell surface molecules CD25, CTLA-4, and GITR are all different therapeutic approaches under investigation. Contributing to the poor success of past approaches is the expression of indoleamine 2,3-dioxygenase 1 (IDO), a tryptophan catabolizing enzyme overexpressed in GBM, and critically involved in regulating tumor-infiltrating Treg levels. Herein, we review the current literature on Tregs in brain cancer, providing a detailed phenotype, causative mechanisms involved in their pathogenesis, and strategies that have been used to target this population, therapeutically.

## Malignant Glioma

Brain tumors fall into different classifications that depend on cellular origin, histological characteristics (i.e., grade), as well as subtype. There are many central nervous system (CNS)-resident cells including neurons, oligodendrocytes, and astrocytes that when transformed, become neuroblastoma, oligodendroglioma, and astrocytoma. Although all of these types of tumors are potentially hazardous, here we focus on malignant glioma, with an emphasis of astrocytoma grade IV [also known as glioblastoma multiforme (GBM)], the most common primary adult brain tumor. GBM is routinely associated with a poor prognosis. Even with an aggressive treatment regimen that involves gross total resection when possible, followed by high dose irradiation and temozolomide (TMZ), the median survival still remains at only 14.6 months (Stupp et al., 2005). Although the annual incidence of GBM is only 3–5 cases per 100,000 individuals, the anatomical localization within the CNS combined with a selectively impermeable blood brain barrier (BBB), results in a lack of many therapeutic agents from entering the tumor effectively. However, today we have a considerably more advanced understanding of the underlying pathogenic mechanisms that lead to gliomagenesis. This rapidly evolving understanding is informing the development of several different therapeutic avenues for future treatment. Of these novel therapeutic strategies, immunotherapy is one of the leading candidates for creating durable and effective outcomes for patients. However, a considerable challenge to developing effective GBM immunotherapy is the complexity of the GBM microenvironment. Within the stroma of the GBM is an intricate but poorly defined meshwork of astrocytoma cells, microglia, astrocytes, pericytes, endothelial cells, as well as many subtypes of leukocytes and hypoxia-induced molecules that collectively contribute to a highly immunosuppressive environment. Converting the glioma microenvironment from one that is tolerant of GBM cells to one that supports immune-mediated tumor rejection is considered to be one of the critical barriers to achieving effective immunotherapy. Of the many immunosuppressive aspects intrinsic to GBM, CD4^+^CD25^+^FoxP3^+^ regulatory T cells (Treg) play a dominant role in deactivating productive anti-GBM immune responses (El Andaloussi and Lesniak, 2006; El Andaloussi et al., 2006; Fecci et al., 2006a).

## Regulatory T Cells

Regulatory T cells, which normally account for only 5–10% of all circulating CD4^+^ T cells, are classically defined as cells that possess the ability to suppress the proliferation of any cytokine-secreting effector T cell [by down-regulating IL-2 and/or interferon-gamma (IFN-γ) production]. Regulatory T cells constitutively express the nuclear transcription factor, FoxP3, as well as cell membrane-resident interleukin-2 receptor alpha [IL-2Rα (CD25)], cytotoxic T lymphocyte antigen-4 (CTLA-4), glucocorticoid-induced tumor necrosis factor (TNF) receptor (GITR), and TNF receptor superfamily member, OX40 (CD134). Under normal physiological conditions, Treg function to maintain tolerance to both host and foreign antigens, resulting in the inhibition of autoimmunity and contribution to the resolution of productive effector T cell responses. In contrast, Treg deregulation, either in the form of loss or gain of function, as well as the depletion or accumulation of cells, contributes to autoimmune and carcinogenic outcomes, respectively (Bennett et al., 2001; Curiel et al., 2004).

The balance between the recruitment and functional state of CD8^+^ cytotoxic T cells (Tc), CD4^+^ conventional T cells (Tconv), and CD4^+^CD25^+^FoxP3^+^ regulatory T cells (Treg) is responsible for maintaining tolerance to self, while also responding to pathogenic challenges arising from foreign bacteria and viruses or endogenous stimuli in the form of cancer. An imbalance in this carefully articulated balance can lead, and sometimes promote, maladaptive reactions to both intrinsic and extrinsic pathogens. This may take the form of autoimmunity, sepsis, chronic inflammation, allergies/asthma, infection, or tumorigenesis. With regard to the latter outcome, the collective action of Tc and Tconv is thought to be overcome by malignancy-induced immunosuppression. Although there are many cellular players involved in suppressing the effector immune response, the hyperactivation and expansion of Treg appears to play a dominant role in inhibiting Tc and Tconv through both cell-contact dependent and -independent processes.

## Regulatory T Cell Differentiation

Regulatory T cells are divided into two subsets based on origin: natural Treg (nTreg) that develop in the thymus and inducible Treg (iTreg) that arise by the induction of FoxP3 in CD4^+^FoxP3^−^ Tconv that have already gone through positive and negative selection in the thymus and emigrated into the periphery (Curotto de Lafaille and Lafaille, 2009; Josefowicz and Rudensky, 2009; Bilate and Lafaille, 2012). During thymic differentiation, Tc and Tconv cell fate is regulated by T cell receptor (TCR) signal strength and duration (Germain, 2002; Singer et al., 2008). Similarly, Treg selection is mediated by a tightly controlled but poorly defined range of TCR affinity and avidity, typically somewhere between the level required for positive selection and the level needed to delete self-reactive effector T cells. Utilizing transgenic mice that possess a fixed TCR-β, direct sequence analysis has defined that the Treg TCR repertoire is very diverse with minimal overlap of TCR repertoire when compared to FoxP3^−^ Tconv (Hsieh et al., 2004; Pacholczyk et al., 2006). Importantly, retroviral transfer of Treg vs. naïve CD4^+^ TCR-α libraries into RAG^−/−^ TCR transgenic T cells showed that the Treg TCR repertoire exhibits increased self-reactivity, based on the ability of Treg TCR-expressing RAG^−/−^ T cells to expand and induce wasting disease in lymphopenic mice, when compared to naïve CD4^+^ Tconv TCR-expressing RAG^−/−^ T cells. T cells retrovirally transduced with Treg TCR also proliferate *in vitro* in response to autologous splenic antigen presenting cells (APC) (as well as in response to invariant chain-deficient APC, which primarily present endogenous protein-derived peptides that are ubiquitously synthesized) in contrast to T cells transduced with non-Treg TCR. Collectively, these data suggest that Treg TCR recognize ubiquitously presented self-antigens (Hsieh et al., 2004).

Physiologically (i.e., *in vivo*), there is minimal TCR recognition overlap between thymic-born and peripherally induced Foxp3^+^ cells, as well as when comparing peripherally induced FoxP3^+^ and Foxp3^−^ cells (Hsieh et al., 2006). In support of this suggestion is data from a mouse model encoding a transgenic TCR-specific for a pancreatic antigen showing that TCR-α chain utilization from both thymic and peripheral Tconv was distinct from the TCR-α chains isolated from Foxp3^+^ Treg (Wong et al., 2007). Furthermore, *in vitro*-generated Treg with TCR stimulation combined with TGF-β and IL-2 exposure, appear to be genetically distinct from *in vivo*-isolated Treg from Foxp3-GFP mice, even though a significant number of genes are shared by both Treg subsets (Haribhai et al., 2011). Interestingly, this study also found that both nTreg and iTreg are required for protection from lymphoproliferative disease, suggesting distinct but complementary roles for the two Treg subsets. Collectively, these data indicate that the majority of Treg are thymic in origin, with specific and distinct requirements for iTreg generation and non-overlapping immunosuppressive roles. A detailed comparison elucidating the differences between nTreg and iTreg is listed in Table 1.

**Table 1:** **Characteristics that distinguish nTreg from iTreg**.

Characteristic	Natural Treg	Induced Treg
Anatomical site of maturation	Thymus	Secondary lymphoid organs/tissue sites of inflammation
Co-stimulation	CD28 and CTLA-4	CTLA-4
Cytokine requirement	IL-2, TGF-β (?)	TGF-β, IL-2, Retinoic Acid
Transcription factors required for development	FoxP3	FoxP3, Ahr
Stability	+++	+
TCR-specificity	Self-antigens (primarily)	Foreign antigens (primarily)
General shared markers	Foxp3, CD25, GITR, CTLA-4	Foxp3, CD25, GITR, CTLA-4
Cell-specific markers	Helios, Nrp1, PD-1, Swap70	Dapl1, Igfbp4
Mechanism of suppression	Cell-contact dependent	Cytokine-dependent (?)
IL-6 can block suppressor activity	Yes	No

*Hsieh et al. (2004), Horwitz et al. (2008), Quintana et al. (2008) Rubtsov et al. (2010), Thornton et al. (2010) Bilate and Lafaille (2012), Verhagen et al. (2013)*.

Although TCR signaling is required for Treg development, the TCR signal, alone, is not sufficient for inducing FoxP3 expression and downstream Treg lineage commitment. Interesting work has recently shown that expression of the TGF-β enhancer, CNS1, is critical for the downstream commitment of iTreg, but not nTreg (Samstein et al., 2012). The lack of iTreg led to increased fetal resorption and placental leukocyte infiltration in allogeneic, but not syngeneic hosts, further indicating a physiological complementarity of the nTreg and iTreg subsets. Moreover, while it has been known for some time that CD28 plays a critical role in the negative selection of Tconv and induction of FoxP3 in thymocytes, recent work has now shown that CTLA-4 also plays a key role in antigen specificity of both nTreg and Tconv (Verhagen et al., 2013). Whether CTLA-4 plays a similar role in iTreg generation has yet to be investigated.

Aside from the differences in nTreg and iTreg function and development, critical differences exist in the regulation of FoxP3 between mice and humans (Ziegler, 2006). Human T cells express two isoforms of FoxP3; the murine FoxP3 ortholog, as well as a splice variant lacking exon 2 (Allan et al., 2005). Another difference in the regulation of Foxp3 expression between mice and humans was found through analysis of stimulated CD4^+^CD25^−^ cells. Stimulation of human CD4^+^CD25^-^ cells using CD3 and CD28 mAbs results in the detectable expression of FoxP3 by 24 h, with a peak in FoxP3 expression at 72 h following the initial stimulus (Walker et al., 2003). In contrast, the CD4^+^CD25^−^ mouse cells do not result in the induction of FoxP3 expression (Fontenot et al., 2003; Hori et al., 2003), suggesting that the temporary induction of FoxP3 is linked to TCR stimulation, alone, in human-, but not mouse-Treg. Another important consideration is that the function of the thymus changes dramatically over a similar period of time between mice and humans. In mice, the naïve T cell pool is sustained throughout the lifetime of the animal by continued thymic production, whereas in humans, the naïve T cell pool is sustained almost entirely through T cell division in the periphery due to the eventual involution of the thymus in adulthood (de Braber, Immunity 2012). Thus, studying the *in vivo* differences between nTreg and iTreg in mice may not fully recapitulate the physiological characteristics relevant to humans.

## Natural and Induced Treg Subsets in Cancer

A consistent finding between previous studies demonstrates that tumors recruit FoxP3^+^ Treg and that this accumulation tends to be progressive, depending on tumor grade (El Andaloussi and Lesniak, 2007; Quezada et al., 2011; deLeeuw et al., 2012; Savage et al., 2013). For the majority of cancers, the accumulation of Treg is associated with an impaired anti-tumor immune response (Onizuka et al., 1999; Shimizu et al., 1999; Turk et al., 2004). In these pre-clinical investigations, the elimination of CD25^+^ Treg results in CD8^+^ T cell-mediated rejection of tumors from various models. Whether tumor-infiltrating Treg are thymic or peripheral in origin remains a subject of open and active study. There is ample evidence supporting the hypothesis that tumors convert CD4^+^FoxP3^−^ (Tconv) into CD4^+^FoxP3^+^ (iTreg) by tumor-derived signals, while others suggest that nTregs are recruited and/or expanded by the tumor (Nishikawa et al., 2003; Curiel et al., 2004; Valzasina et al., 2006; Liu et al., 2007b; Hindley et al., 2011). The roles of the two Treg subsets in cancer are not necessarily mutually exclusive. In a study using a model of B cell lymphoma and hemagglutinin, it was found that both nTreg and *de novo*-produced iTreg combinatorially contribute to the Treg pool in the context of a tumor (Zhou and Levitsky, 2007). Ultimately, the ratio of nTreg to iTreg in a specific cancer may simply depend on anatomical location, grade of tumor, and cellular origin. However, determining this type of Treg may result in the ability to develop more selective Treg-depleting therapies.

## Tregs and Glioma

**Figure 1:**
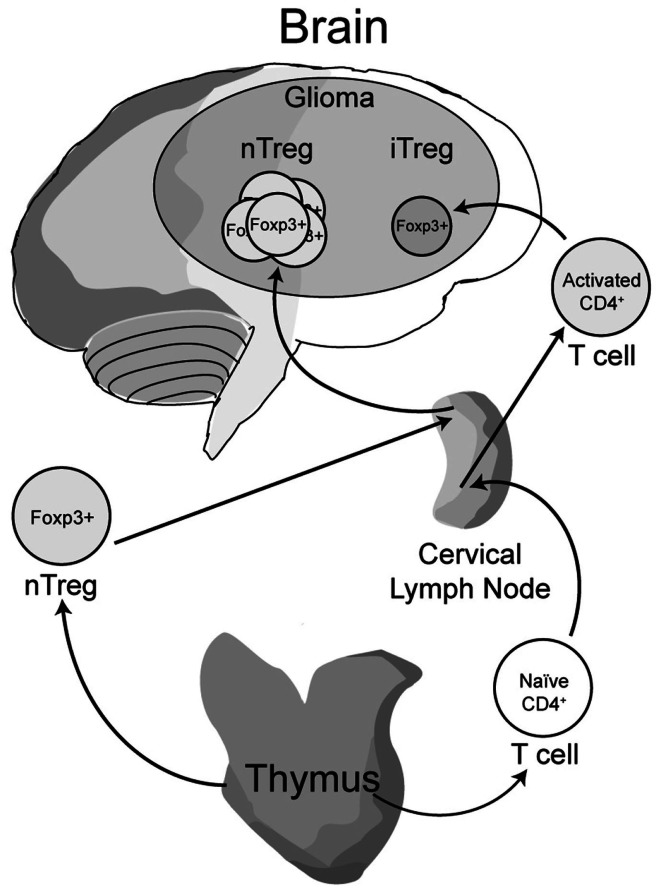
**A model depicting the development, maturation and recruitment of regulatory T cells (Treg) in glioma-bearing hosts**. Thymus-derived (natural) nTreg are the predominant resident in glioma, when compared to the tumor-induced iTreg. Although both Treg subsets express FoxP3, these subsets respond to different classes of antigens and possess different epigenetic stability with regard to FoxP3-regulated immunosuppressive control and cytokine function. Moreover, nTreg primarily recognize self-antigens, while iTreg predominantly recognize tumor-associated antigens that are not normally endogenously expressed. Upon infiltration and/or expansion in(to) the glioma, Treg promote tumor formation by suppressing CD8^+^ cytotoxic T cell-mediated tumor rejection. (Floess et al., 2007; Thornton et al., 2010; Wainwright et al., 2011).

Early work from our laboratory and independent groups identified a progressive increase in the numbers of CD25^+^FoxP3^+^ Treg with WHO grade II, III, and IV (GBM) astrocytoma, respectively, either in the peripheral circulation or within the tumor of human resected gliomas (Fecci et al., 2006a; El Andaloussi and Lesniak, 2007; Heimberger et al., 2008a). Subsequent observations found that thymus-derived nTreg, rather than glioma-induced iTreg, represent the predominant population of CD4^+^FoxP3^+^ T cells within brain tumors (Wainwright et al., 2011) (Figure 1). Our group compared normal mice with brain tumors to those that had been previously thymectomized, with or without administration of the Treg-depleting CD25 mAb. Thymectomy, alone, resulted in a significant decrease of tumor-infiltrating Treg, supporting the hypothesis that glioma is predominantly infiltrated by thymus-derived Treg. Furthermore, combining thymectomy with CD25 mAb further decreased tumor-infiltrating Treg levels, although this was not statistically significant from the thymectomy alone group. In support of these data, we reported the expression of Helios, an Ikaros-family transcription factor shown to be expressed exclusively by nTreg and not iTreg (Thornton et al., 2010), to be expressed by ∼90% of all brain tumor-resident Treg. To confirm that this finding was not specific to mice, we also showed that Helios^+^ Tregs predominate in human GBM as well. Our finding that nTreg are the predominant Treg subtype in brain tumors has recently been supported in other cancer models using updated and refined methodology of detection (Hindley et al., 2011; Malchow et al., 2013). Therapeutically, these results imply that future Treg-depleting strategies by targeting nTreg based on their unique antigen-specific TCR repertoire may be more selective and therefore (potentially) possess fewer side-effects. This relies on the hypothesis that nTreg depletion is associated with more effective anti-glioma effector response coincident with a greater survival advantage, which is now the suggested dogma (El Andaloussi et al., 2006; Grauer et al., 2008; Banissi et al., 2009; Maes et al., 2009; Wainwright et al., 2011).

Independent of the importance related to Treg origin is the finding that soluble factors originating from GBM promote the *in vitro* expansion of Tregs while simultaneously inducing the expression of pro-apoptotic genes in Tconv (Crane et al., 2012). The presence of tumor cell-conditioned medium also causes conventional CD4^+^ T cells to transiently upregulate the expression of Foxp3 and TGF-β. Interestingly, 10 days of co-culturing CD4^+^ T cells in GBM-conditioned media was long enough to return TGF-β and FoxP3 levels to a similar level found in Tconv. This temporary induction of the Treg phenotype in Tconv, *in vitro*, is in line with our *in vivo* finding that brain tumor-infiltrating Tregs are primarily thymus-derived, rather than converted from a Tconv population.

## Treg Trafficking to GBM

Chemotaxis of leukocytes occurs, in part, through the interaction of chemokines interacting with cognate chemokine receptors. This interaction represents a highly promiscuous relationship and reflects the interaction between many different chemokine receptors that possess redundant roles for recognizing multiple cognate chemokines (Mailloux and Young, 2010; Zlotnik and Yoshie, 2012). Once such interaction is between the chemokine, CCL22, and its cognate chemokine receptor, CCR4, which is expressed on Tregs and has been implicated in Treg recruitment to tumors using multiple models (Curiel et al., 2004; Miller et al., 2006; Jacobs et al., 2010). In glioma, ∼74% of Treg isolated from the peripheral blood of GBM patients express CCR4, which is significantly increased when compared to the ∼43% of Treg expressing CCR4 in healthy (control) patients (Jordan et al., 2008). These data suggest that some soluble factor(s) originating from the GBM primes Treg to induce or upregulate CCR4. Coincidently, GBM-resected specimens have previously been shown to produce CCL2 and CCL22, both of which are chemokines that attract CCR4-expressing Treg (Sebastiani et al., 2001; Jordan et al., 2008). These findings collectively suggest that one novel strategy for therapeutic intervention may involve the inhibition of Treg trafficking to the GBM, as has been shown to be an effective approach in other models of cancer (Pere et al., 2011). However, to determine the physiological significance of inhibiting the CCL22-CCR4 axis in human GBM, it may be prudent to first test this as a proof-of-concept in mouse GBM models, which has yet to be performed. Critical points to address in these pre-clinical studies include the degree of redundancy between Treg-recruiting chemokines, whether selective chemokine blockade unintentionally disrupts Tc and Tconv homing to the glioma, as well as the clinically relevant consideration: how chemokine neutralization affects the Treg population already within the tumor bed.

## TGFβ, Tregs, and Glioma

TGF-β production by glioma represents a complex aspect regulating Tregs in brain tumors. Since glioma expresses high levels of select TGF-β isoforms, combined with the role of TGF-β in converting Tregs *in vitro*, one might expect an increased level of iTreg in brain tumors (Kaminska et al., 2013). However, one possible explanation for the finding that most Treg are thymus-derived (Wainwright et al., 2011), is the contribution of the stroma in determining whether Treg are recruited rather than converted from Tconv. Given the microenvironment of the brain, including the unique contribution of the BBB, unique mode of lymphatic drainage and highly immunosuppressive environment, even under normal conditions, the mechanisms regulating Treg accumulation in brain tumors may be independent of the TGF-β signaling pathway. However, it is important to note that TGF-β neutralization leads to a decrease in the level of brain tumor-infiltrating Tregs (Ueda et al., 2009) suggesting that this cytokine somehow plays a role in Treg recruitment and/or expansion.

## Regulatory T Cells and Antigen Specificity

The antigen specificity of tumor-infiltrating Tregs is a complex issue and is under current investigation by many laboratories, including our own. Since nTreg primarily recognize self-antigens, whereas iTreg most frequently recognize foreign antigens, the question of which antigens are being recognized by tumor-infiltrating Treg is important, given that nTreg appear to dominate in many tumor environments; including GBM. Moreover, the enrichment of nTreg may reflect an insufficient level of TCR stimulation required for peripheral Treg induction in response to “foreign” antigens. It is also possible that iTreg promoting cytokines are not present at the requisite levels in the tumor microenvironment (Savage et al., 2013). Although the identification of a Treg antigen in the context of malignancy remains to be a challenging task, a central question regarding tumor-infiltrating nTreg antigen specificity has recently been addressed. In a recent study by Malchow et al. (2013), a single TCR (designated MJ23) was found to be over-represented in the tumor-infiltrating Treg population using a transgenic mouse model of prostate cancer. Interestingly, the identified TCR was specific to an antigen expressed in normal prostate tissue. After generating a mouse with the transgenic MJ23 TCR, the authors demonstrated that it was sufficient to drive Treg development in the thymus in an Aire-dependent manner. These results have important implications for Treg antigen specificity in glioma. Since a majority of Treg in the GBM are nTreg, these GBM-infiltrating Treg may recognize self-antigens that are derived from the CNS. However, whether this paradigm holds true in experimental GBM models and in patients remains a tantalizing consideration for further study. Ultimately, investigating the antigen specificity of Treg in glioma is a way to increase our understanding of Treg biology for the design of selective therapeutic strategies to counter Treg-induced immunosuppression in brain tumors.

## Tryptophan Catabolism and Brain Tumors

**Figure 2:**
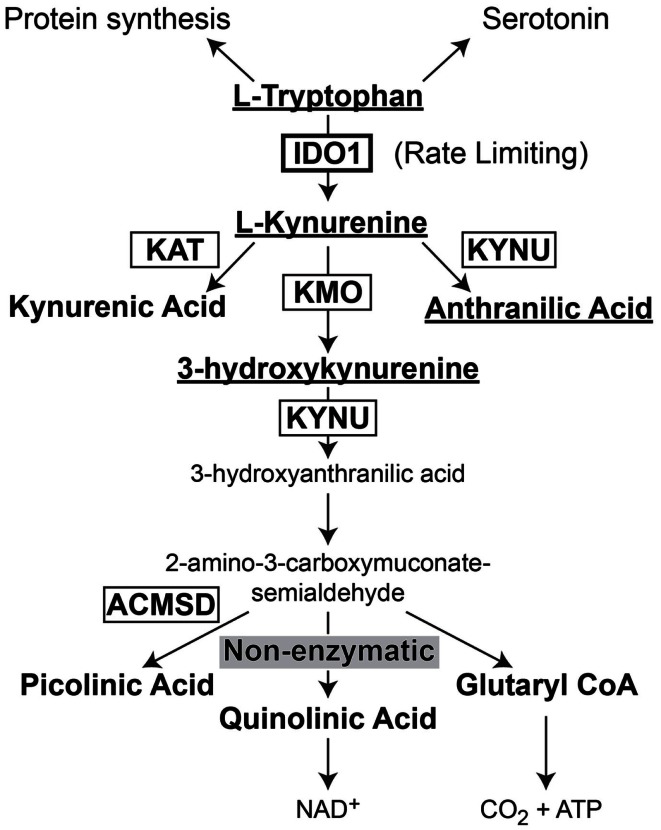
**Schematic representation of l-Tryptophan consumption in the body**. l-tryptophan is an essential amino acid that is utilized during protein synthesis. In the presence of tryptophan hydroxylase and the co-factor, iron (not shown), l-tryptophan is converted to the neurotransmitter, serotonin. However, in the presence of indoleamine 2,3-dioxygenase 1 (IDO), l-tryptophan is converted to l-kynurenine. l-kynurenine can then be converted to kynurenic acid, 3-hydroxykynurenine, or anthranilic acid via kynurenine aminotransferase (KAT), kynurenine 3-monooxygenase (KMO), and kynureninase [KYNU (also known as l-kynurenine hydrolase)], respectively. 3-hydroxyanthranilic acid is converted by 3-hydroxyanthranilic acid oxygenase (not shown) to 2-amino-3-carboxymuconate-6-semialdehyde. This downstream catabolite can further be catabolized to the final metabolic products of picolinic acid via picolinic carboxylase [also known as 2-amino-3-carboxymuconate-6-semialdehyde-dacarboxylase (ACMSD)], quinolinic acid through a non-enzymatic process or glutaryl CoA through a series of oxido-reductive reactions.

Indoleamine 2,3-dioxygenase 1 (IDO) is the rate-limiting enzyme that mediates catabolism of the essential amino acid, tryptophan, to downstream catabolites leading to end products of picolinic acid and NAD^+^, as well as CO_2_ and ATP (Figure 2). Additional enzymes that have a tryptophan catabolizing capability include indoleamine 2,3-dioxygenase 2 (IDO2) and tryptophan 2,3-dioxygenase (TDO). Notably, TDO has recently been highlighted to have an association between upregulated expression in patient glioma specimens and an overall decrease in survival (Opitz et al., 2011). However, here we focus on the function of IDO, its relevance in cancer and inflammation and how it regulates Treg in GBM.

## IDO and Tryptophan Catabolism

Indoleamine 2,3-dioxygenase 1 was first identified to be involved in tryptophan catabolism in 1975 by a group that provided the first evidence of catalytic activity and biological function (Hayaishi et al., 1975; Hirata et al., 1975). Shortly therein, the group identified superoxide anions to be a critical component required for IDO activity (Taniguchi et al., 1977) and that Fe^2+^ was also required for this complex assembly (Hirata et al., 1977). By 1978, it was suggested that IDO was associated with inflammatory processes, since it was induced upon the exposure of mice to *E. Coli*-derived lipopolysaccharide in the lungs of mice (Hayaishi and Yoshida, 1978; Yoshida and Hayaishi, 1978). Importantly, this mechanism possessed a quick turnover based on a peak in tryptophan catabolism at ∼24 h and decreasing back to baseline by 6 days post-treatment. Collectively, these data demonstrated IDO to be induced by inflammation, superoxide anion-dependent, and possessing both a heme group and catalytic activity for the pyrrole ring of indoleamine-containing compounds.

## Interaction between IDO, Leukocytes, and Tumor Cells

Several pro-inflammatory factors have been identified that induce IDO expression in human peripheral blood cells including IFN-α, IFN-β, IFN-γ, and LPS (Carlin et al., 1987, 1989); although other individual and combinatorial pro-inflammatory agents are capable of regulating IDO expression and activity as well. At approximately the same time, it was found that lung cancer patients bearing malignant tumors had a 20-fold higher level of IDO, when compared to patients bearing benign lesions (Yasui et al., 1986). *In vitro* exposure of lung slices to IFN-γ further demonstrated that this cytokine played a critical role in the induction of IDO; although IFN-α was also capable of inducing IDO expression, albeit less potently. Interestingly, early experiments analyzing the allogeneic anti-tumor immune response found that IDO was induced in tumor cells when injected into an allogeneic-, but not syngeneic-hosts (Yoshida et al., 1988). When allogeneic and syngeneic cells were mixed and then injected, intraperitoneally (i.p.), the induction of IDO occurred in both cell types, suggesting a global inflammatory promoter (such as IFN-γ). However, it is important to note that only the allogeneic cells were rejected under these conditions and not syngeneic cells, even though both cell types were infiltrated by mononuclear cells (Yoshida et al., 1988). Thus, IDO induction appeared to be regulated by pro-inflammatory factors and is involved the anti-tumor immune response.

In 1995, IFN-γ-induced IDO activity was demonstrated in three different types of ovarian xenografts in nude mice that lack endogenous T cells (Burke et al., 1995). In all three tumor models, l-tryptophan was significantly depleted, commensurate with the presence of l-kynurenine, which was also found in the surrounding tissues. Interestingly, *in situ* hybridization demonstrated IDO expression in all areas of the tumor, not just in the surrounding peripheral cells. Also notable was that IDO expression remained elevated in tumor cells, even when tryptophan levels had already returned to normal levels, suggesting some level of post-transcriptional regulation of the expression. By the year 2000, a correlation between IDO-expressing dendritic cells and T cell function was established (Hwu et al., 2000). It was found that both CD40L and T cell-expressed IFN-γ could regulate IDO expression by *in vitro*-cultured DCs. Functionally, the impairment in T cell proliferation induced by DC-expressed IDO could be reversed when 1-dl-methyltryptophan (1-MT), a pharmacological inhibitor of IDO, was added to the DC-T cell co-cultures. Further studies showed that IL-6 played a critical role in reversing the tolerogenic functions of *in vitro*-cultured CD8^+^ tolerogenic DCs by decreasing IFN-γ receptor and that this activity was correlated to the decreased ability for tryptophan degradation (Grohmann et al., 2001). Shortly thereafter, it was shown that mice pre-immunized with IDO-transfected cells could significantly inhibit the allogeneic T cell response in adoptively transferred cells (Mellor et al., 2002).

## Inhibition of IDO as a Potential Tumor Therapy

The first evidence that inhibiting IDO could be utilized as a therapeutic modality against tumors came in 2002, when Friberg et al. (2002) showed that mice bearing Lewis lung carcinoma and administered 1-MT demonstrated slower tumor growth, when compared to mice not administered the IDO inhibitor. Further evidence showed that many different types of human tumors express high levels of IDO expression including 100% of prostatic, colorectal, pancreatic, cervical, and endometrial carcinomas, with 90% of GBM specimens expressing variable levels of IDO (Uyttenhove et al., 2003). It was also demonstrated that pre-immunized mice were unable to reject tumors that were IDO^+^, suggesting that IDO overrides the anti-tumor immune response. This effect could be partly reversed when the pre-immunized mice were co-administered 1-MT.

Independent of tumor-derived IDO, it has been shown in mouse models that DC residing in tumor-draining lymph nodes also possess potently immunosuppressive properties (Munn et al., 2004) which include the activation of mature Treg (Sharma et al., 2007). Functionally, these plasmacytoid DCs (pDC) are rendered ineffective when genetically deficient for- or pharmacologically inhibited for-IDO activity, strongly suggesting that IDO is a critical requirement for the immunosuppression induced in pDC. Independent support for this hypothesis was demonstrated when investigators showed that the CD200 engagement with CD200R on pDC induces and/or regulates IDO expression (Fallarino et al., 2004). However, the relationship between CD200 expression in tumor-draining lymph nodes has yet to be established. *In vitro*-based work has also identified a 2-step requirement for induction and enzymatic activation of IDO in DC (Braun et al., 2005). The induction step requires exposure to prostaglandin E2 (PGE_2_), while the activation step requires signaling through either the tumor necrosis factor receptor or a toll-like receptor agonist. These data may be particularly relevant when considering DC-based immunotherapy protocols, since limiting IDO expression would be a desirable characteristic for maximal therapeutic efficacy.

Although 1-MT has long been used as a potent inhibitor of IDO enzymatic activity, the search for other, potentially more effective and/or combinatorially applied agents continues. Accordingly, 3-(4-morpholinyl)sydnonimine, a peroxynitrite generator, significantly inhibits IDO activity without affecting expression levels (Fujigaki et al., 2006). Specifically, nitration of Tyr15 was the most important factor related to inhibiting IDO activity. In a separate *in vitro* study, H_2_O_2_ showed potent inhibitory properties against IDO activity by oxidation of cysteine residues to sulfinic and sulfonic acids (Poljak et al., 2006). Intriguingly, celecoxib, a cyclooxygenase 2 inhibitor has been shown to decrease the expression of IDO in a spontaneous mammary gland tumor model, *in vivo* (Basu et al., 2006). With regard to the various IDO inhibitors, tonic regulation of IDO expression and inhibition is likely to depend on dosage of the agent. One example of this is the demethylating drug, Zebularine. At low-doses, Zebularine increases the immunogenicity of tumor cells, while high doses decreases immunogenicity; the latter of which is dependent on increased IDO expression (Liu et al., 2007a). From an immunotherapeutic perspective, it would be desirable to increase the immunogenicity of tumors to increase the likelihood of antigen-specific Tc-mediated tumor cell killing. Finally, it is important to note that stereoisomers of different compounds may possess greatly different effects in future IDO inhibitory strategies. For example, 1-MT is found as 2 stereoisomers; levorotary (L) and dextrorotary (D). Importantly, while L1-MT appears to significantly inhibit IDO1 activity, D1-MT is a relatively inefficient inhibitor of IDO1 and rather, appears to effectively inhibit IDO2 (Lob et al., 2009). This highlights how subtleties in enzymatic modulation can vary widely using virtually identical compounds, while also raising questions as to why D1-MT appears to play a stronger immunotherapeutic role, when compared to the actions of L1-MT (Hou et al., 2007).

## IDO, Tregs, and Glioma

Early work in human astrocytes demonstrated that these cells are very sensitive to the effects of IFN-γ by upregulating the enzyme, IDO and the production of NAD (Grant et al., 2000). Further studies showed that IFN-γ also stimulated IDO expression in transformed astrocytes *in vitro* (Grant and Kapoor, 2003; Miyazaki et al., 2009) and *in vivo* (Uyttenhove et al., 2003). Although this potent IFN-induced IDO expression may play a protective anti-viral role under certain conditions (Adams et al., 2004), it also appears to play a maladaptive role in the context of brain cancer.

Recent work from our laboratory has demonstrated that the upregulation of IDO mRNA in resected glioma specimens is associated with a significant decrease of overall survival in patients with glioma (Wainwright et al., 2012). This correlation between increased IDO and decreased survival has been confirmed at the protein level, independently (Mitsuka et al., 2013). Our investigation found that the expression of IDO by brain tumor cells, rather than peripheral cells (i.e., astrocytes, microglia, pDC, etc.), mediated tumorigenesis. This was supported by the finding that IDO-competent tumors accumulate immunosuppressive Tregs in both IDO-competent and -deficient mice (Wainwright et al., 2012). In contrast, IDO-deficient tumors fail to support significant Treg expansion in both IDO-competent and -deficient mice. Importantly, the IDO-regulated Treg expansion was associated with a significant decrease in overall survival, when compared to mice bearing IDO-deficient brain tumors. Notably, the beneficial effect on survival was not solely due to the lack of Treg recruitment to the glioma since mice lacking any major T cell subset (i.e., Tc and/or Tconv) failed to support long-term survival, even in the absence of IDO expression by brain tumors.

The IDO-mediated Treg accumulation in brain tumors predominantly reflects an expansion of thymus-derived, rather than tumor-induced Tregs (Wainwright et al., 2011). This implies that one of the primary effects of IDO is to induce and/or increase the chemokines that attract Tregs. Supporting this hypothesis is the finding that IDO^+^, but not IDO^−^ DCs express the Treg-recruiting chemokine, CCL22 (Onodera et al., 2009). Coincidently, GBM cells resected from patients have been shown to express the chemokine, CCL22 (Jordan et al., 2008). Whether IDO expression directly mediates the upregulation of CCL22 in GBM cells has yet to be determined. Since the principal function of IDO is suggested to be enzymatic in nature, it is possible that IDO catabolizes tryptophan to kynurenine and that a downstream kynurenine catabolite acts as a co-factor to increase CCL22. This may occur through the recently discovered interaction between kynurenine and aryl hydrocarbon receptor (Ahr) (Mezrich et al., 2010), based on previous data showing that Ahr cooperates with RelB to affect chemokine production (Vogel et al., 2007a,b,c). Although CCL22 was not analyzed in these studies, the potential effect of Ahr on CCL22 transcription is likely to be contextual and therefore, dependent on multiple factors from the tumor microenvironment.

**Figure 3:**
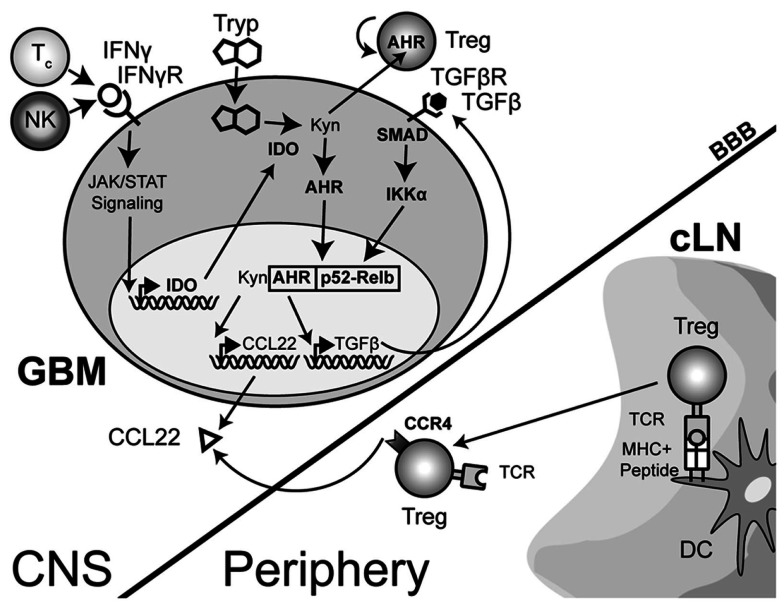
**A theoretical model depicting the process of regulatory T cell (Treg) recruitment and expansion in brain tumors**. The interaction of Treg with cervical (draining) lymph node (cLN)-resident dendritic cells (DC) through the T cell receptor (TCR)-peptide/major histocompatibility complex (MHC) II activates and epigenetically stabilizes the expression of CCR4-expressing Treg. These recently stimulated Treg then emigrate from the cLN into the circulation where they can respond to the gradient of the Treg-recruiting chemokine, CCL22, being produced by the glioma. Within the glioma, innate natural killer (NK) cells that initially respond to the tissue disruption and inflammatory signals produced by the tumor are later joined by antigen-specific CD8^+^ cytotoxic T cells (Tc); both of which produce the pro-inflammatory cytokine, interferon-gamma (IFN-γ). This cytokine acts on the glioma-expressed, IFN-γR, resulting in downstream Janus-kinase/signal transducer and activator of transcription (JAK/STAT) activity that subsequently induces indoleamine 2,3-dioxygenase 1 (IDO). IDO mediates the enzymatic conversion of l-tryptophan to l-kynurenine. The latter metabolite interacts with the cytoplasmically localized, aryl hydrocarbon (AHR). This interaction drives the localization of this hormone-like receptor into the nucleus. Simultaneously, TGF-β signaling (via glioma-expressed TGF-βR engagement) drives SMAD-induced IKK-α phosphorylation, leading to p52 (RelB) nuclear translocation. Nuclear Ahr and p52 interact, leading to a unique transcriptional response further driving IDO expression (not shown), as well as CCL22 and TGF-β chemokine and cytokine expression, respectively. Independently, and due to the permeable nature of the downstream catabolite, l-kynurenine, Treg proliferation occurs. However, while many components of this paradigm have been shown to occur in disparate cell types, the comprehensive scheme proposed has yet to be shown in glioma, specifically. (Hotfilder et al., 2000; Vogel et al., 2007b,c; Jordan et al., 2008; Mezrich et al., 2010; Opitz et al., 2011; Pallotta et al., 2011; Wainwright et al., 2011, 2012).

The complexity of IDO signaling may allow it to directly activate CCL22 transcription through a slightly different mechanism in addition to the one described above. Recent work has now shown that aside from the enzymatic function of IDO, there is a distinct signaling component due to the interaction of IDO with TGF-β-induced SHP1 and SHP2 (Pallotta et al., 2011). The interaction leads to the phosphorylation of IKKα and the release of RelB, nuclear translocation and subsequent downstream transcriptional effects. In the context of a brain tumor, it is therefore possible that the TGF-β-induced SHP proteins interact with IFN-γ-induced IDO, resulting in the release of RelB, interaction with Ahr and downstream effects that include the activation of CCL22 transcription (Figure 3). However, this hypothesis remains to be tested.

## Therapeutic Targeting of Tregs

**Figure 4:**
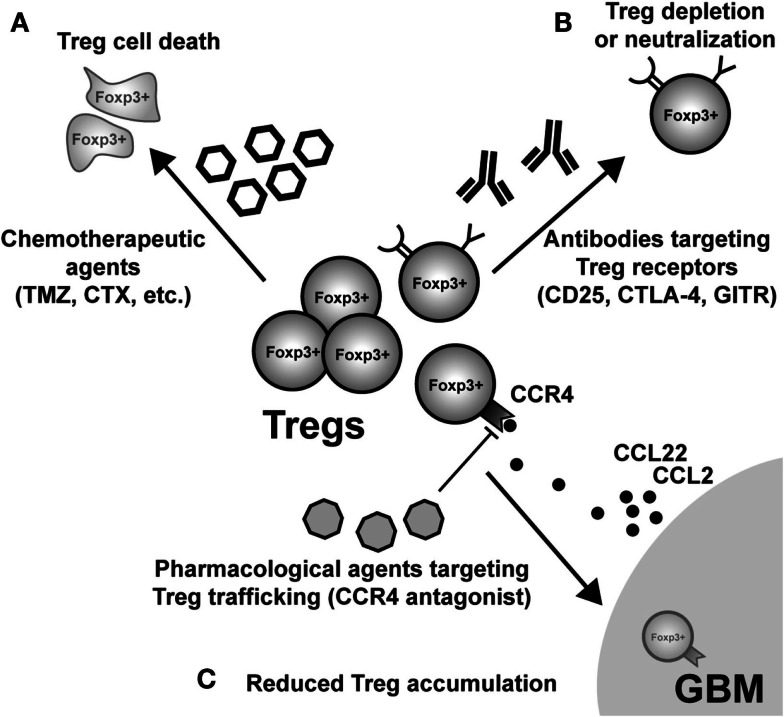
**Possible modes of therapeutic Treg neutralization for the treatment of glioblastoma multiforme (GBM)**. **(A)** Low-dose administration of the chemotherapeutic agents, temozolomide (TMZ), and cyclophosphamide (CTX), have been shown to have a beneficial impact on Treg levels in both patients and animal models of GBM. **(B)** Targeting constitutively expressed receptors on the cell surface of Treg is another way that has demonstrated variable levels of success. IL-2Rα (CD25) targeting with monoclonal antibodies neutralizes and/or depletes Treg, *in vivo*. Similarly, agonistic GITR mAb leads to inhibition of Treg suppressor capability and loss of tumor-homing capacity. In contrast, CTLA-4 mAb appears to inhibit the Treg-mediated induction of indoleamine 2,3-dioxygenase 1 (IDO) in dendritic cells (DC), as well as possesses independent effects on effector T cells. **(C)** Neutralizing GBM-expressed chemokines that attract Treg are an additional mode for potential therapy. However, it is important to note that since most GBM patients will be diagnosed after Treg accumulation has begun to occur, chemokine neutralization may not be a promising approach. Regardless, the physiological (i.e., *in vivo*) potential for this therapeutic effect has to be investigated in glioma. (Fallarino et al., 2003; Dannull et al., 2005; Ko et al., 2005; El Andaloussi and Lesniak, 2006; El Andaloussi et al., 2006; Fecci et al., 2006a, 2007; Jordan et al., 2008; Banissi et al., 2009; Davies et al., 2009; Zhao et al., 2010; Mitchell et al., 2011, 2012; Pere et al., 2011; Fong et al., 2012; Sampson et al., 2012).

Given the pathogenic role that Treg mediate in the context of malignant brain tumors, an obvious therapeutic direction is their selective depletion from both the tumor microenvironment and/or secondary lymphoid tissues, where Tc and Tconv priming and effector function is affected. This highly translational research arm is a very active and dynamic field, with the goal of determining which monotherapy and/or combinatorial therapy will lead to the greatest impact on decreasing Treg numbers and/or function. Below, we highlight some of the most prescient targets leading to the disruption of Treg function and/or depletion (Figure 4). However, it should be noted that in addition to those therapeutic agents and targets described below, far more effective and specific methods are currently being developed.

### Temozolomide

Temozolomide, a second generation DNA alkylating agent, methylates the O^6^ position of guanine causing double stranded DNA cross-linking. The DNA damage results in calcium-dependent apoptosis and autophagy, eventually leading to cell death. TMZ is also reported to activate p53 and p21^WAF1/Cip1^-mediated G2/M cell cycle arrest with subsequent apoptosis or senescence (Nagasawa et al., 2012). Although TMZ is well-tolerated and has an overall beneficial impact on patient survival, it has also been well-described to induce immunosuppression, most often described as various forms of lymphopenia (Lanzetta et al., 2003; Kocher et al., 2005). The association between TMZ and lymphopenia is the preferential depletion of CD4^+^CD25^+^ Treg (Su et al., 2004). Both in human and animal studies of glioma, TMZ has been demonstrated to alter Treg trafficking toward glioma cells, *in vitro*, as well as solid tumors, *in vivo*. (Jordan et al., 2008; Banissi et al., 2009; Kim et al., 2010). Clinically, TMZ appears to be beneficial when combined with a peptide vaccine targeting the epidermal growth factor receptor variant III (EGFRvIII) (Heimberger et al., 2008b). Perhaps the reason this vaccine strategy worked well was that the TMZ was administered to coincide with times points that would target maximal Treg depletion. The strategy of combining TMZ-induced Treg depletion and vaccination is now being in tested in ongoing phase II and III clinical trials. Since TMZ is the current standard of care for glioma patients, manipulating the beneficial TMZ-induced side-effect(s) holds a particular appeal in this patient cohort.

### Cyclophosphamide

Cyclophosphamide, like TMZ, is also an alkylating agent that at high doses, results in potent cytotoxicity and lymphoablation. CTX has been used as an anti-cancer therapy since 1959. However, due to the high cytotoxicity and side-effects, the routine use of CTX in glioma is limited with the exception of administering continuous low-doses (also called metronomic dosing). Metronomic CTX dosing has been shown to have immunostimulatory effects that include the expansion of antigen-specific tumor-reactive T cells, a transient depletion of Treg and the restoration of DC homeostasis (Radojcic et al., 2010). This mechanism has been suggested to occur by the preferential depletion of CD8^+^ lymphoid-resident DC, increased potency of pDC, increased migratory capacity of DC, as well as elevated antigen presentation and cytokine secretion (Nakahara et al., 2010). Ultimately, the metronomic CTX schedule has been shown to result in an anti-tumor immune responses by stimulating the effector arm of the immune response, while simultaneously inhibiting immunosuppression (Langroudi et al., 2010; Sharabi et al., 2010; Zhao et al., 2010).

In a murine model of colon cancer, the combination of IL-12 and CTX eliminates intratumoral Treg and myeloid-derived suppressor cells, while simultaneously inducing pro-inflammatory myeloid cells within the tumor microenvironment, an essential component for facilitating effector T cell infiltration and subsequent tumor rejection (Medina-Echeverz et al., 2011). In support of this approach, PD-1 blockade, low-dose CTX, and combinatorial peptide administration has been shown to synergistically induce a strong antigen-specific immune response by increasing Tc and Tconv infiltration into the malignancy, ultimately leading to potent tumor rejection (Mkrtichyan et al., 2011). Even in a canine model, 15 mg/m^2^/day of CTX leads to a decrease in the total number and frequency of Treg in the peripheral blood, while simultaneously increasing serum IFN-α concentrations (Burton et al., 2011; Mitchell et al., 2012). Moreover, in 12 patients with treatment-refractory metastatic breast cancer receiving single-agent CTX, there was a significant initial reduction in circulating Treg by more than 40% (*p* = 0.002), although this decrease was transient, since Treg levels returned to pre-CTX treatment levels due to increased proliferative activity (Ge et al., 2012). Moreover, while Treg suppressor activity was maintained at normal levels, the overall Treg depletion led to an increase in breast tumor-reactive T cells (*p* = 0.03) that remained at high levels throughout treatment; correlating with disease stabilization (*p* = 0.03) and overall survival (*p* = 0.027). Depleting Treg has been attempted, clinically, in several human cancers including ovarian cancer (Vermeij et al., 2012), cervical cancer (Peng et al., 2013), renal cancer (Huijts et al., 2011), melanoma (Berd et al., 1990), and glioma (Plautz et al., 2000). However, the optimal timing of this strategy still requires better definition with regard to anatomy and malignant progression.

### STAT3

Signal transducer and activator of transcription 3 (STAT3) controls the transcription of several genes in response to cytokines and growth factors. IL-2, a cytokine critical for the maintenance of Treg *in vivo*, contributes to Foxp3 expression in human CD4^+^CD25^+^ Tregs via STAT3 and STAT5 (Zorn et al., 2006). However, it should be noted that while STAT3^-/-^ progenitors show no sign of Treg developmental block, STAT5α/β^−/−^ lymphoid progenitors possess a significant inhibition toward developing into thymus-derived Treg (Yao et al., 2007). Thus, the regulation of STAT3-induced Treg development appears to require additional co-factors (i.e., STAT5) for normal maturation.

STAT3 regulates the expression of TGF-β and IL-10, crucial cytokines that contribute to the presence of tumor-associated Treg (Kinjyo et al., 2006). Interestingly, tumor-bearing mice with STAT3^−/−^ hematopoietic cells possess a significant reduction in the number of tumor-infiltrating Treg (Kortylewski et al., 2005). Thus, developing agents that inhibit STAT3 is a rapidly emerging goal for eventually designing a new class of compounds that inhibit Treg development, function and/or tumor-infiltration. In the context brain metastasis arising from melanoma cells, the novel STAT3 inhibitor, WP1066, reverses immune suppression through the inhibition of FoxP3 induction in peripheral T cells and down-regulation of Foxp3 expression in nTreg (Kong et al., 2009). Based on this and other promising pre-clinical studies demonstrating the beneficial effects of STAT3 inhibition (Bill et al., 2010; Hatiboglu et al., 2012; Liu et al., 2013), STAT3 inhibitors are now being tested in clinical trials for advanced solid tumors.

### CD25

Several strategies have been attempted for depleting Treg based on the constitutively expressed cell surface marker, CD25. In the context of hematological malignancy, a phase I study using LMB-2, a CD25 mAb conjugated to truncated *Pseudomonas* exotoxin, was found to elicit a promising clinical response (Kreitman et al., 2000). However, in the setting of metastatic melanoma, despite inducing a transient decrease in Treg *in vivo*, LMB-2 administration failed to augment the immune response to cancer vaccination and patients neither experienced an objective beneficial response nor severe side-effect in the form of autoimmunity (Powell et al., 2007).

The recombinant IL-2-diphtheria toxin conjugate, DAB(389)IL-2 (also known as denileukin diftitox and ONTAK) was designed for use as a Treg-depleting agent. However, there are mixed reports regarding its ability to successfully deplete Treg and stimulate the anti-tumor immune response (Attia et al., 2005a; Dannull et al., 2005; Mahnke et al., 2007). In non-Hodgkins lymphoma patients, although the combination of denileukin diftitox with rituximab decreased the number of CD25^+^ T cells, denileukin diftitox significantly increased the toxicity of the combination without an improvement in response rate or time to progression (Ansell et al., 2012).

In the setting of malignant glioma, CD25 mAb has been used in several studies as a means to deplete Treg. In an experimental model of glioma, GL261 cell-based brain tumor-bearing mice pre-treated with CD25 mAb lived significantly longer than those bearing tumor and receiving control IgG antibody (El Andaloussi et al., 2006). The mechanism of action was associated with a decrease in the frequency of brain tumor-infiltrating CD4^+^CD25^+^ T cells, while simultaneously eliminating their suppressor activity. The inhibition of Treg function permits enhanced lymphocyte proliferation and IFN-γ production with as much as 80% lysis of glioma cells *in vitro*. When combined with DC immunization, CD25 mAb elicits tumor rejection in 100% of challenged mice (Fecci et al., 2006b). Furthermore, using GL261 cell-based brain tumor-bearing mice treated with both intraperitoneally and intracranially administered CD25 mAb results in long-term survival and complete tumor rejection, when compared to the systemic administration of CD25 mAb alone. (Poirier et al., 2009). Accordingly, the depletion of Treg with CD25 mAb strongly enhances the efficacy of DC vaccination, although CD25 mAb had an anti-tumor effect independent of the DC vaccination response as well (Maes et al., 2009). Importantly, DC vaccination is required to protect animal models from intracranial tumor re-challenge, since no long-term protection was observed in animals that had initially received CD25 mAb alone.

Treg depletion functions differently based on immunocompetent and lymphopenic contexts, as well as when it is given in relation to the overall tumor burden. Accordingly, CD25 mAb in normal mice decreases intratumoral Treg and contributes to tumor rejection in small tumors, but is less effective in large established tumors and also disrupts the effector arm of the immune response (Curtin et al., 2008). In contrast, in lymphodepleted hosts, CD25 mAb decreases Tregs without impairing effector T cell responses (Mitchell et al., 2011). In a randomized placebo-controlled pilot study, combinatorial administration of humanized CD25 mAb, Daclizumab, with peptide vaccination against the EGFRvIII and lymphodepleting TMZ safely and selectively depleted Treg in patients with GBM (Sampson et al., 2012). Moreover, it was reported that Daclizumab treatment was well-tolerated with no symptoms of autoimmune toxicity and a significant decrease in the frequency of circulating Treg when compared to saline-treated controls.

### CTLA-4

CTLA-4 is a constitutively expressed cell surface molecule on Treg. Like its closely resembling ligand, CD28, CTLA-4 also binds to the co-stimulatory molecules, CD80 and CD86 on APC, acting as a powerful negative regulator of T cell activation (McCoy and Le Gros, 1999) via the induction of indoleamine 2,3-dioxygenase and/or TGF-β (Fallarino et al., 2003; Rudd, 2008). In both humans and in mouse models, Treg from malignant gliomas have been shown to express high levels of CTLA-4 (El Andaloussi and Lesniak, 2006; El Andaloussi et al., 2006). CTLA-4 is not only associated with glioma progression and prognosis, the CTLA-4 A49G polymorphism might also be a potential clinically relevant biomarker for distinguishing individuals with a high risk for developing glioma (Wu et al., 2011). In a human study of DC vaccines, it was found that monitoring the changes in Treg frequency and dynamic expression of the negative co-stimulatory molecules on peripheral blood T cells, before and after DC vaccination, may predict survival (Fong et al., 2012). In the GL261 mouse model of glioma, combining Treg depletion with CTLA-4 neutralization boosts glioma-specific Tc and Tconv effector T cell responses, while also increasing anti-glioma IgG2_A_ antibody titers; ultimately resulting in complete tumor rejection (Grauer et al., 2007). Using the same model, vaccination with granulocyte-macrophage colony-stimulating factor (GM-CSF)-expressing whole glioma cell vaccination followed by CTLA-4 blockade has been demonstrated to significantly improve survival (Agarwalla et al., 2012). In the SMA-560 mouse model of glioma, neutralization of CTLA-4 with monoclonal antibody, 9H10, confers a long-term survival benefit in 80% of treated mice with re-establishment of normal CD4 counts concomitant with a decreased Treg fraction. Interestingly, treatment benefits appeared to be primarily mediated through the CD4^+^CD25^−^ T cell population rather than the Treg population, as CD4^+^CD25^−^ T cells from treated mice showed improved proliferative responses and resistance to Treg-mediated suppression, whereas Treg from the same mice remained “tolerogenic” and displayed no defect in suppressor function (Fecci et al., 2007). Based on these and other promising pre-clinical studies, humanized CTLA-4 mAb has now been successfully tested in clinical trials for the treatment of metastatic melanoma (Mathew et al., 2013; Wilgenhof et al., 2013). However, results from late phase clinical trials studying the therapeutic effects of this antibody for treating patients with malignant glioma have yet to be reported (Phan et al., 2003; Attia et al., 2005b; Maker et al., 2005) and must be considered in the context of the potent neurological side-effects that have been previously reported (Bot et al., 2013).

### GITR

Gluococorticoid-induced TNFR-related protein (GITR, also known as TNFRSF18), a type I transmembrane protein with homology to other TNF receptor family members such as OX40, CD27, and 4-1BB, is normally expressed at very low levels on resting Tc, low levels on Tconv and at constitutively high levels on Treg (Cohen et al., 2010). Currently, there are competing theories regarding the impact of the GITR-GITRL interaction on Treg. While there is evidence to suggest that this interaction renders responder T cells more susceptible to suppression by Treg, it has also been shown that GITR signaling in Treg, directly, inhibits the ability to mediate suppression of responder T cells (Shevach and Stephens, 2006). In a mouse model of fibrosarcoma, T cell stimulation by agonistic GITR mAb attenuated Treg-mediated suppression and enhanced tumor-killing by Tc and Tconv cells via increased secretion of IFN-γ. This worked synergistically with co-administration of CTLA-4, but not with CD25 mAb (Ko et al., 2005) reinforcing the hypothesis that while both GITR and CD25 mAb inhibit Treg-mediated suppressor activity as a primary mechanism of promoting tumor immunity, CTLA-4 mAb independently contributes to tumor rejection by directly acting on effector T cells. In the B16 cell-based mouse model of melanoma, the agonist GITR mAb, DTA-1, induces regression of small established tumors in mice. Although DTA-1 neither altered systemic Treg frequencies nor their intrinsic suppressor activity, intratumoral accumulation of Treg was significantly impaired, resulting in a greater Teff:Treg ratio, thereby enhancing tumor-specific CD8^+^ T cell activity (Cohen et al., 2010).

Independently, we have shown that IDO-competent brain tumors promote tumor-infiltrating Treg to significantly upregulate the expression of GITR, when compared to IDO-deficient tumors (Wainwright et al., 2012). Interestingly, this effect is locally regulated since Treg in IDO-competent tumors have upregulated levels of GITR when compared to those Treg in draining cervical lymph nodes and/or spleen. Ultimately, the overabundance of GITR on glioma-infiltrating Treg may provide the necessary avidity for future therapeutic antibody approaches that selectively target intratumoral – rather than systemic – Treg inhibition. Currently, a humanized GITR mAb (TRX518), developed by Toleryx, Inc., is in a Phase 1 safety and tolerability dose-escalation clinical trial for late stage (III and IV) melanoma patients with unresectable tumor, although this agent has not yet been investigated for primary brain tumor patients.

### OX40

OX40 (also known as CD134), another member of the TNF receptor family, is expressed on naive Tregs and transiently upregulated following TCR stimulation. OX40 stimulation in Treg using agonistic antibodies inhibits the capacity to suppress, thereby restoring effector T cell proliferation, IL-2 gene transcription and cytokine production (Valzasina et al., 2005). Using a mouse tumor model, it has been shown that agonistic OX40 mAb, but not CD25 mAb, induces tumor rejection in 80% of mice. OX40-mediated functional inactivation of Treg recruits nearby DC, promoting the induction of an adaptive immune response (Piconese et al., 2008). Additionally, combinatorial therapy using CTX and OX40 mAb provides potent anti-tumor immunity resulting in the regression of established melanoma in a B16 cell-based model. Within the tumor, combinatorial therapy induces a profound depletion of Treg depletion accompanied by an influx of effector T cells leading to a favorable Teff:Treg ratio (Hirschhorn-Cymerman et al., 2009). In a brain tumor model, mice bearing GL261 cell-based glioma were susceptible to the treatment with OX40 mAb and tumor regression was dependent on the participation of both Tc and Tconv cells (Kjaergaard et al., 2000). Similarly, mice bearing intracranial GL261 cell-based brain tumors and treated with a combination of OX40 mAb, local cranial radiotherapy, as well as intrasplenic vaccination with DC demonstrated the complete regression of tumor resulting in long-term survival (≥120 days) with no evidence of tumor recurrence and resistance to further intracranial tumor challenge (Kjaergaard et al., 2005). Importantly, OX40 mAb-mediated therapy is currently being tested in a Phase 1/2 trial for patients with metastatic melanoma (NCT01689870), as well as in a Phase 1 trial for patients with advanced forms of cancer (NCT01644968) although it has yet to be initiated for primary brain tumors, specifically.

### FoxP3

Targeting the constitutively expressed receptor, CD25, to neutralize Treg is limited by the challenge of the transient expression on Tc and/or Tconv during activation-induced upregulation, including those vaccine-associated effector T cells that carry out anti-tumor responses. As of today, Foxp3, a nuclear transcription factor required for generating nTreg, is the only gene product known to be (almost) exclusively expressed by Treg in mice. Notable exceptions to this rule are Tr1 and Th3 CD4^+^ regulatory T cells that professionally express IL-10 and TGF-β, respectively, but do not express FoxP3.

Vaccination of mice with FoxP3 mRNA-transfected DC elicits a robust FoxP3-specific Tc response that contributes to vaccine-associated protective immunity. As might be implied by the more promiscuous expression of CD25, relative to FoxP3 on or in Treg, respectively, CD25 mAb and FoxP3 vaccination have slightly different effects in tumor-bearing mice. While CD25 mAb depletes Treg systemically, FoxP3 vaccination leads to Treg depletion intratumorally, with sparing of Treg in the periphery (outside of the tumor (Nair et al., 2007). This disparity may reflect the ability of vaccine-programed effector T cells to differentially sense aberrantly programed Treg, as we and others have demonstrated that intratumoral Treg are phenotypically distinct (Gounaris et al., 2009; Cohen et al., 2010; Wainwright et al., 2010, 2012; Blatner et al., 2012), vs. the effects of an antibody that lacks such sophistication.

Another strategy for targeting FoxP3 in Treg utilizes a synthetic peptide, P60, that binds directly to FoxP3. P60 enters Treg and inhibits FoxP3 nuclear translocation, decreasing the ability to suppress the transcription of NF-κB and NFAT. When P60 was administered to BALB/c mice and immunized with the Tc epitope, AH1, from CT26 tumor cells, immune-mediated protection against tumor implantation occurred (Casares et al., 2010). Although this approach has shown some promising results, pre-clinically, this strategy has yet to be tested in the context of patients with cancer.

## Conclusion

Regulatory T cells are a highly important lineage of immune cells that maintain tolerance to self, provide regulatory stability during the resolution of inflammation and expand as a population during pregnancy to suppress the potential spontaneous T cell-mediated rejection due to paternally derived fetal alloantigens. Although beneficial under normal circumstances, pathological Treg responses can promote autoimmunity or malignant transformation in the absence or overabundance of function/accumulation, respectively. Thus, understanding factors that regulate Treg suppressor activity, cytokine production, homing, expansion, contraction, induction, conversion, TCR reactivity, and interaction with other cells is a critically relevant area of investigation.

The level of Treg accumulation in malignant astrocytoma is progressive; increasing with tumor grade and maximal in GBM (El Andaloussi and Lesniak, 2006). The depletion of Treg in models of malignant brain tumors extends survival and ameliorates disease, depending on timing, tumor size, and dosage of the depleting agent. Given the constitutively high expression of certain molecules on the Treg cell surface (i.e., CTLA-4, GITR, and CD25), depleting, and/or neutralizing these cells with CTLA-4-, GITR-, and CD25-mAbs is an attractive therapeutic modality. However, these approaches tend to have many side-effects, additional targets (i.e., Tc and Tconv), as well as a relatively high degree of toxicity to patients. Thus, additional approaches are needed to address these concerns.

Recent identification of Treg in brain tumors as predominantly arising from a thymus-derived origin (Wainwright et al., 2011) may enlighten future investigation with regard to delineating antigen specificity, clonality of this cellular pool, as well as epigenetic programing (given the high stability of nTreg relative to iTreg). If it is found that the tumor-infiltrating nTreg are primarily clonal in nature and therefore, expand from few Treg progenitors, antigen-specific therapies may have a higher chance of becoming highly effective, given the smaller TCR repertoire that will be required to target against. However, if it is determined that Treg in GBM arise from a more heterogenous population, and therefore originating from a high amount of TCR-distinct clones, then an antigen-specific therapy for depleting Treg may be a less attractive approach, given the potentially enormous amount of variation between Treg in tumors, as well as the additional variation that may arise between individuals. Regardless of either outcome, the TCR repertoire in GBM-infiltrating Treg is currently unknown and therefore an important future research endeavor to pursue. It is equally important to keep in mind that nTreg depletion via TCR-specific targeting may lead to more effective tumor rejection, while simultaneously increasing bystander damage to CNS-resident astrocytes (and any other cells co-expressing the TCR-specific peptide/MHC II complex that the Treg is reactive to). Theoretically, this would be due to the loss of Treg-mediated tolerance against astrocytes. However, the overall risk due to the loss of dominant tolerance to a single antigen is likely to play a minimal role in causing autoimmune pathology, since nTreg are likely to react with many astrocyte-specific antigens.

The recent finding that nTreg accumulation in brain tumors is dependent on the expression of IDO represents an exciting new direction for Treg research in neuro-oncology. IDO is an attractive target for therapeutic consideration given its minimal expression in normal CNS-resident neurons and glia, versus its high expression in GBM. It is important to appreciate that *in vitro*-cultured GBM cells express negligible IDO levels normally (Miyazaki et al., 2009). In contrast, the exposure of pro-inflammatory cytokines, such as IFN-γ, rapidly induces IDO expression and tryptophan catabolic activity in GBM cells, *in vitro*. However, whether this simple induction of IDO, *in vitro*, fully reflects the pro-tumorigenic activity, *in vivo*, is doubtful, given that this enzyme also possesses the ability to regulate downstream signaling events. Moreover, our recent observation demonstrating IDO promoting gliomagenesis by increasing the recruitment of Treg to brain tumors (Wainwright et al., 2012) must be interpreted carefully. This observation was based on the orthotopic GL261 cell-based model whereby shRNA was used to permanently knockdown IDO expression in implanted tumors. However, by virtue of intracranial implantation, the BBB was temporarily disrupted at a time that inflammation was ectopically induced. This inflammation is likely to have co-induced the upregulation of damage associated molecular pattern receptors (DAMP) and downstream signaling cascades (Topfer et al., 2011), as well as a presumed release of CNS-resident antigen to the cervical draining lymph nodes. This collective action may have contributed to an artificially induced anti-tumor response that aided brain tumor rejection in the absence of IDO expression. Although we did verify a decreased level of glioma-resident Treg in the GFAP:(12)V-Ha-Ras transgenic glioma model (Shannon et al., 2005) that was backcrossed to a globally IDO^−/−^ background, this did not distinguish the contribution of glioma-expressed- and peripherally expressed-IDO to CNS-resident Treg. To better understand the role of IDO in brain tumors under normal conditions, we have now created a transgenic model of glioma that is selectively deficient for IDO only in cells capable of forming astrocytoma by backcrossing a tamoxifen-induced GFAP-Cre driven high grade astrocytoma mouse model (Chow et al., 2011) with floxed IDO mice. This new mouse model will allow us to study the contributions of tumor-derived versus peripheral sources of IDO with regard to Treg recruitment, the anti-tumor immune response, as well as overall impact on survival.

Aside from IDO1, IDO2, and TDO are also tryptophan catabolic molecules co-expressed by glioma. Coincidently, the upregulation of TDO in glioma is strongly associated with decreased overall survival in patients (Opitz et al., 2011). However, the roles of IDO2 and TDO in the regulation of Treg recruitment to glioma has yet to be investigated. It is interesting that clinical trials currently investigating IDO inhibitors as an adjuvant immunotherapy are currently utilizing D1-MT (Table 2), which inhibits IDO2, rather than IDO1 (Lob et al., 2009). However, this finding is controversial, since a separate study found that L1-MT, rather than D1-MT, is a better inhibitor of IDO2 tryptophan catabolic activity (Qian et al., 2012). Regardless, it was also shown that even in the presence of a high concentration of 1-MT, IDO2-induced T cell proliferative growth arrest could not be inhibited. Collectively, these data suggest that, in addition to IDO1, IDO2 and TDO may also be high-impact targets for investigating their contribution to modulating Treg levels, as well as overall future therapeutic possibilities for glioma patients.

**Table 2:** **Ongoing clinical trials using 1-MT as an adjuvant immunotherapy**.

Title	Status	Identifier	Goal
IDO inhibitory study for relapsed or refractory solid tumors (D1-MT)	Terminated	NCT00739609	Determine the safety and efficacy of D1-MT in patients with recurrent or refractory solid tumors. Establish the toxicities of D1-MT and define any dose-limiting toxicities.
D1-MT in treating patients with metastatic or refractory solid tumors that cannot be removed by surgery	Recruiting patients	NCT00567931	Phase I trial to study effects and best dose of D1-MT in treating patients with metastatic or refractory solid tumors that cannot be removed by surgery.
D1-MT and Docetaxel in treating patients with metastatic solid tumors	Recruiting patients	NCT01191216	Phase I trial to study the effects and best dose for giving D1-MT and Docetaxel together in treating patients with metastatic solid tumors.
Vaccine therapy in treating patients with metastatic breast cancer	Recruiting patients	NCT01042535	Randomized Phase I/II trial to study the side-effects and best dose of giving vaccine therapy and to assess the effectiveness in treating patients with metastatic breast cancer.
Study of chemotherapy in combination with IDO inhibitor in metastatic breast cancer	Ongoing, not recruiting patients	NCT01792050	To compare the effects, good and/or bad, of standard of care therapy (Docetaxel) with or without the co-administration of D1-MT.
Phase II study of Sipuleucel-T and Indoximod for patients with refractory metastatic prostate cancer	Recruiting patients	NCT01560923	Randomized Phase II, double blind, multi-institutional study of Indoximod or placebo after the completion of standard of care Sipuleucel-T in men with asymptomatic or minimally symptomatic metastatic prostate cancer that is hormone refractory.

In summary, we highlight Treg as critical cells involved in suppressing the anti-glioma immune response. This mechanism involves the co-inhibitory ligand CTLA-4, is therapeutically modulated with Treg-depleting CD25 mAb and Treg function-modulating GITR agonistic mAb. We also highlight the tryptophan catabolizing enzyme, IDO1, as a critical modulator of Treg recruitment and/or expansion to/within the glioma, as well as raise the possibility that enzymes with similar catabolic activity, IDO2 and TDO, may be attractive future targets for immunotherapeutic consideration. With these insights in mind, Treg immunomodulation as a means to increase GBM immunogenicity appears to be a rapidly developing approach.

## Conflict of Interest Statement

The authors declare that the research was conducted in the absence of any commercial or financial relationships that could be construed as a potential conflict of interest.
